# Microstructural changes in the trigeminal nerve of patients with episodic migraine assessed using magnetic resonance imaging

**DOI:** 10.1186/s10194-020-01126-1

**Published:** 2020-05-29

**Authors:** Tiffani J. Mungoven, Noemi Meylakh, Kasia K. Marciszewski, Vaughan G. Macefield, Paul M. Macey, Luke A. Henderson

**Affiliations:** 1 Department of Anatomy and Histology, F13, University of Sydney, Sydney, NSW 2006 Australia; 2 Baker Heart & Diabetes Institute, Melbourne, Australia; 3 UCLA School of Nursing and Brain Research Institute, University of California, Los Angeles, California 90095 USA

**Keywords:** Trigeminal root entry zone, Nerve volume, Diffusion tensor imaging, Fractional anisotropy, Mean diffusivity

## Abstract

**Background:**

There is histological evidence of microstructural changes in the zygomaticotemporal branch of the trigeminal nerve in migraineurs. This raises the possibility that altered trigeminal nerve properties contribute to migraine pathophysiology. Whilst it is not possible to explore the anatomy of small trigeminal nerve branches it is possible to explore the anatomy of the trigeminal root entry zone using magnetic resonance imaging in humans. The aim of this investigation is to assess the microstructure of the trigeminal nerve in vivo to determine if nerve alterations occur in individuals with episodic migraine.

**Methods:**

In 39 migraineurs and 39 matched controls, T1-weighted anatomical images were used to calculate the volume (mm^3^) and maximal cross-sectional area of the trigeminal nerve root entry zone; diffusion tensor images were used to calculate fractional anisotropy, mean diffusion, axial diffusion and radial diffusion.

**Results:**

There were significant differences between the left and right nerve of controls and migraineurs with respect to volume and not cross-sectional area. Migraineurs displayed reduced axial diffusion in the right nerve compared to the left nerve, and reduced fractional anisotropy in the left nerve compared to left controls. Furthermore, although there were no differences in mean diffusion or radial diffusion, regional analysis of the nerve revealed significantly greater radial diffusion in the middle and rostral portion of the left trigeminal nerve in migraineurs compared with controls.

**Conclusions:**

Migraine pathophysiology is associated with microstructural abnormalities within the trigeminal nerve that are consistent with histological evidence of altered myelin and/or organization. These peripheral nerve changes may provide further insight into migraine pathophysiology and enable a greater understanding for targeted treatments of pain alleviation.

## Background

Histological evidence of structural abnormalities in the trigeminal nerve in individuals with episodic migraine has been recently reported in only a single study to date [1]. More specifically, migraineurs displayed discontinuous and non-uniform proportions of the myelin sheaths along the length of isolated zygomaticotemporal nerve branches. Neurofilaments appeared discontinuous and poorly associated with the myelin sheaths, suggesting axonal abnormalities. Whilst this is the only investigation to explore the anatomy of the trigeminal nerve in migraineurs, it raises the possibility that altered trigeminal nerve properties contribute to migraine pathophysiology. Indeed, we and others have used human brain imaging techniques to non-invasively show alterations in the trigeminal nerve anatomy in other chronic orofacial pain conditions such as trigeminal neuralgia and painful trigeminal neuropathy [2–5].

Whilst it is not possible to use human magnetic resonance imaging (MRI) techniques to reliably explore the structure of the zygomaticotemporal division of the trigeminal nerve, it is possible to reliably explore the anatomy of the trigeminal nerve root entry zone as it lies in the pontine cistern. In a previous study we used T1-weighted anatomical and diffusion weighted images to explore the trigeminal nerve root entry zone anatomy, and found that trigeminal neuralgia subjects displayed a significant decrease in nerve volume, whereas painful trigeminal neuropathy subjects displayed a significant increase, although neither group displayed a change in free-water diffusion between left and right nerve group means [5]. These differences likely reflect differing peripheral mechanisms and potentially different degrees of degeneration of myelinated and unmyelinated axons proximal to the injury site [6, 7]. Furthermore, although the peripheral nerve divisions are not separable, it has been shown that C-fibers lie primarily within the caudal aspect and *A*δ-and *A*β- fibers in the rostral aspect of the trigeminal root entry zone [8]. As a consequence, we are in a position to examine parts of the trigeminal nerve that relate to the processing of noxious compared to non-noxious somatosensory inputs.

The existence of anatomical changes in the trigeminal nerve of migraineurs may indicate that differences in nerve structure contribute to pain modulation in migraine. It has been suggested that the microstructural nerve alterations measured using MRI techniques may be indicative of processes such as axonal loss and demyelination [3, 9–11]. Demyelination of the trigeminal nerve may lead to points along the nerve in which action potentials are generated ectopically and spontaneously, and may also lead to ephaptic transmission between axons. Axon-axon communication where calcitonin gene-related peptide (CGRP) containing C-fibers modulate adjacent *A*δ-sensory nerves at the nodes of Ranvier, which are unmyelinated gaps in the myelin sheath in the trigeminal system, suggest the occurrence of such interactions along the sensory fibers [12]. This may have implications for the alteration of sensory processing, neural sensitization and the subsequent degradation of the myelin sheath in migraineurs. Demyelination would likely be evidenced by an increase in microstructural variability of the nerve [13–15]. Whilst changes in trigeminal nerve firing may not be enough to generate a migraine itself, they may increase the propensity of either an external stimulus or a change in brain sensitivity to trigger a migraine attack. This observation hints at the underlying pathophysiology of migraine, which may be amendable to specifically targeted therapeutics that could effectively alleviate migraine pain.

The aim of this investigation is to use T1-weighted anatomical and diffusion weighted images to assess the structure of the left and right trigeminal nerve in individuals with episodic migraine. We hypothesize that, analogous to trigeminal neuropathy, the volume and cross-sectional area of the trigeminal nerve will be larger in migraineurs compared with controls. Furthermore, given the histological evidence of altered myelin organization, we hypothesize that fractional anisotropy, a measure of free water movement directionality, will be reduced and mean diffusivity, a measure of the average molecular motion independent of any tissue directionality, will be increased in the trigeminal nerve root entry zone of migraineurs compared with controls. Finally, given that noxious afferents lie primarily in the caudal part of the trigeminal nerve root entry zone, we hypothesize that these differences in diffusivity will be more prominent caudally.

## Methods

### Subjects

Thirty-nine subjects with migraine (29 females; mean ± SEM age, 29.97 ± 1.55 years) and 39 pain-free controls (23 females; mean ± SEM age 30.70 ± 2.01 years) were recruited from the general population using an advertisement. There were no significant differences in age (t-test, *p* > 0.05) or gender composition (χ^2^ test, p > 0.05) between the control and migraine group. Migraine subjects were diagnosed according to the International Classification of Headache Disorders (ICHD), 3rd edition, sections 1.1 and 1.2 [16]. Ten migraineurs reported aura associated with their migraines and the remaining 29 reported no aura. All migraine subjects were scanned during the interictal period (the pain- and symptom- free period between migraine attacks), at least 72 h after and 24 h prior (subsequently verified with a headache diary) to a migraine event.

The exclusion criteria for migraineurs included the presence of any other pain condition or neurological disorder. Controls were exempt from the study if they had a family history of migraines, currently used analgesic medications or if they suffered from any other pain condition or neurological disorder. All participants were subject to standard MRI exclusion criteria. No migraineur was excluded based on their medication use and no migraine or control subject had an incidental neurological finding that resulted in their exclusion from the study. All migraine subjects indicated the pain intensity during their most recent migraine (6-point visual analogue scale; 0 = no pain, 5 = most intense imaginable pain) and specified the distribution of their pain commonly experienced during a migraine attack on a face map. Furthermore, each subject described the qualities of their migraine pain as well as any current treatments and medications used to prevent or abort a migraine once initiated. This study was approved by the Institutional Human Research Ethics Committee at the University of Sydney and informed written consent was obtained for all participants in accordance with the Declaration of Helsinki. Data from 30 of the 39 migraineurs were used in previous investigations [17–20].

### MRI acquisition

Subjects lay supine on the bed of a 3-T MRI scanner (Phillips, Achieva) with their head immobilized in a 32-channel head coil. In each subject a high-resolution 3D T1-weighted anatomical image set covering the entire brain was collected (turbo field echo; echo time = 2.5 ms, repetition time = 5600 ms, flip angle 8^0^, voxel size 0.8 × 0.8 × 0.8 mm). In addition, a high-resolution diffusion tensor image (DTI) image set covering the entire brain was collected using a single-shot, multi section, spin-echo echo-planar pulse sequence (repetition time = 8788 ms; flip angle = 90^0^, matrix size 112 × 112, field of view 224 × 224 mm, slice thickness = 2.5 mm, 55 axial slices). For each slice, diffusion gradients were applied along 32 independent orientations with *b* = 1000 s/mm^2^ after the acquisition of *b* = 0 s/mm^2^ (b0) images. The b0 value reflected the strength and timing of the gradients used to generate DTI; the high b value of 1000 s/mm^2^ generated images with stronger gradients and faster slew rates.

### MRI analysis

#### Trigeminal nerve volume and maximum cross-sectional area analysis

**Fig. 1:**
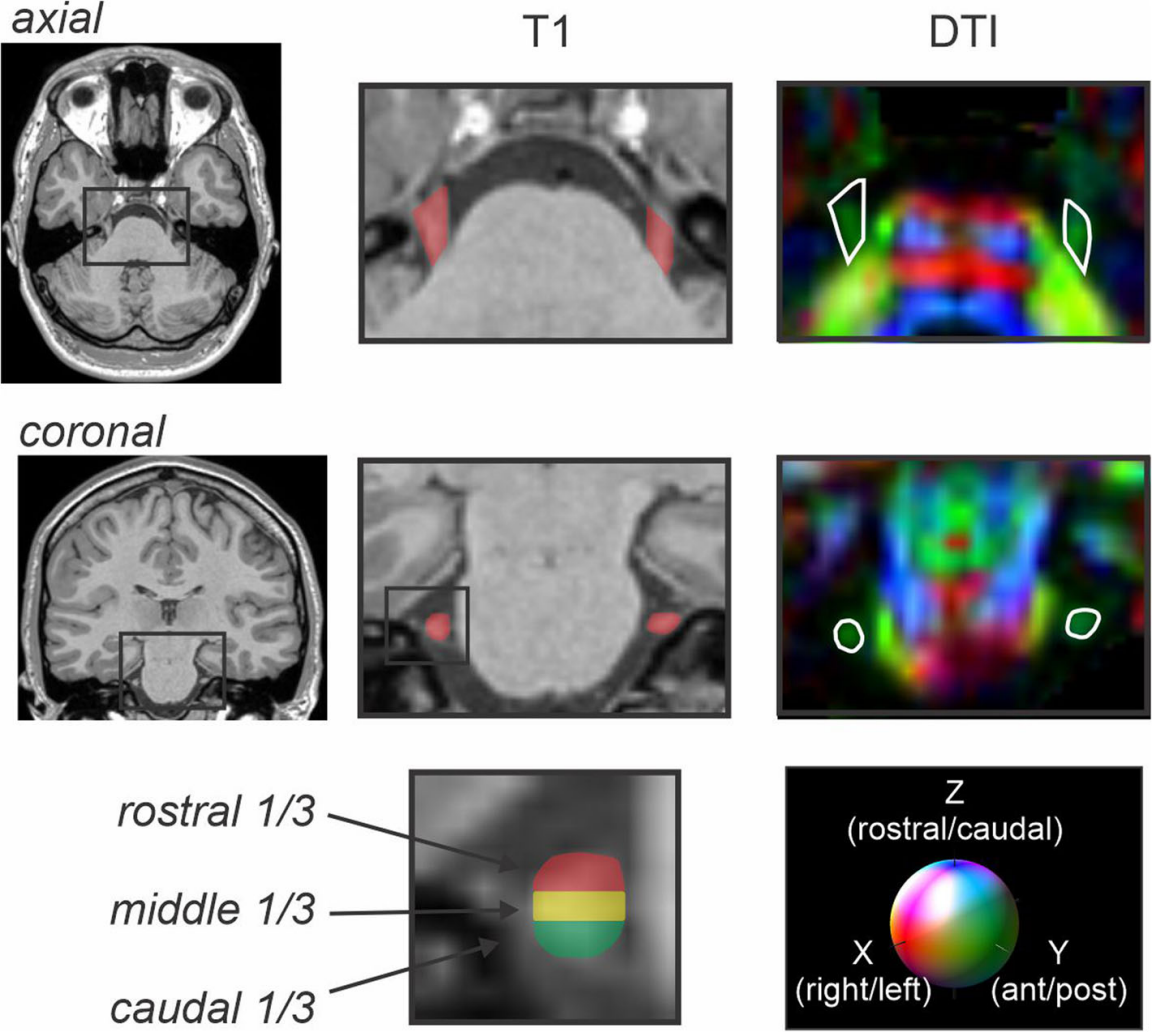
Axial and coronal T1-weighted anatomical images and corresponding diffusion tensor (DTI) images showing the trigeminal nerve root entry zone in a single subject. The DTI image is color-coded for direction of greatest water movement. The outline of the trigeminal nerve region used for total nerve analysis is also shown in red shading on the T1-weighted anatomical image and outlined in white on DTI images. To the right is an example of the regions selected for the rostral (red), middle (yellow) and caudal (green) third of the nerve at the maximal cross-sectional area

Using Statistical Parametric Mapping (SPM) version 12 software [21], the T1-weighted anatomical image from each subject was resampled at a higher resolution of 0.3 × 0.3 × 0.3 mm in order to improve visualization of the trigeminal nerve and to reduce the inclusion of surrounding tissue and cerebrospinal fluid. Using the resampled images, a tracer (author TJM) blinded to the group assignment, outlined the left and right trigeminal nerves within the root entry zone using MRIcroN software. The root entry zone is the section of the trigeminal nerve that lies within the pontine cistern, i.e. from where it emerges from the pons, to the point at which it exits the pontine cistern anteriorly (Fig. 1). All three orthogonal planes were used in defining the nerve on both sides, with the axial plane being the first plane used, followed by coronal and sagittal views. A volume of interest (VOI) encompassing the entire trigeminal root entry zone was anatomically defined using the Duvernoy Brainstem Atlas [22] and manually traced on each subject’s T1-weighted image, using MRIcron [23]. To assess intra-tracer and inter-tracer reliabilities, author TJM retraced a subset of right trigeminal nerves in 10 random subjects (5 controls, 5 migraineurs) and a second tracer (NM), also blinded to group assignment and the earlier investigator’s results, repeated the tracings on the same 10 trigeminal nerves. Inter- and intra-tracer reliabilities were calculated using Cronbach’s Alpha and Dice similarity coefficients for total volumes. The total volumes (mm^3^) within the isolated nerves were calculated by extracting and averaging the volume from each voxel inside the isolated region of the trigeminal root entry zone in the left and right nerve for each individual control and migraine subject. Furthermore, the cross-sectional volume of the nerve in each coronal slice was calculated and the maximum coronal cross-sectional area value (mm^2^) was determined.

For the control and migraine groups, mean (±SEM) volume and maximum cross-sectional areas were calculated. Significant differences in volume and cross-sectional areas between control and migraine groups were determined using unpaired t-tests (two-tailed, *p* < 0.05), whereas within-group significant differences were determined using paired t-tests (two-tailed, *p* < 0.05). In addition, linear relationships between volume and cross-sectional areas in migraineurs with migraine duration, migraine intensity and migraine frequency were determined (Pearson’s correlation, p < 0.05).

#### Trigeminal nerve diffusion analysis

Using SPM12 software and the Diffusion toolbox [21], all diffusion tensor image sets from each subject were motion corrected, based on b0 images within each series. Using diffusion-weighted images collected from 32 directions and b0 images, the diffusion tensor was calculated from all the images using a linear model [24]. Once the elements of diffusion tensor were calculated, whole-brain maps of fractional anisotropy (FA), mean diffusivity (MD), axial diffusion (AX) and radial diffusion (RD) were calculated. All images remained in native space for these calculations. The DTI images were shifted to the subject’s anatomical space by coregistering the b0 DTI image to the T1-weighted image.

The diffusion images were then resampled to a voxel size of 0.3 × 0.3 × 0.3 mm. Using these resampled images, the left and right trigeminal nerves within the root entry zone were isolated as aforementioned. Using the FA image, all three orthogonal planes were used in defining the nerve and a volume of interest encompassing the entire trigeminal root entry zone was created for each subject. The mean (±SEM) FA, MD, AX and RD values were calculated for the left and right trigeminal nerves in all subjects.

For the control and migraine groups, mean (±SEM) FA, MD, AX and RD values were calculated. Significant differences in all four diffusivity values between control and migraine groups were determined using unpaired t-tests (two-tailed, *p* < 0.05). In addition, linear relationships between diffusivity values in migraineurs with migraine duration, migraine intensity and migraine frequency were determined (Pearson’s correlation, p < 0.05).

To study nerve fiber demography, the trigeminal root entry zone was divided into caudal, middle and rostral thirds (Fig. 1). FA, MD, AX and RD values were then calculated for each of these three regions for the left and right nerves in each control and migraine subject. For the control and migraine groups, mean (±SEM) FA, MD, AX and RD values were calculated at each level. Significant differences in diffusion values between groups were determined using unpaired t-tests (two-tailed, *p* < 0.05). Within-group significant differences were determined using paired t-tests (two-tailed, p < 0.05).

## Results

### Migraine characteristics

Migraineurs reported their pain distribution commonly experienced during a migraine attack over the last 12 months as mostly confined to the orofacial region and occasionally the neck. Twenty-one migraineurs reported that their headaches were normally unilateral in nature whereas the remaining 18 migraineurs reported them to be primarily bilateral. Migraine subjects most frequently described their migraine pain as “throbbing,” “pulsating,” and/or “sharp” in nature. They indicated that “stress,” “lack of sleep,” and/or “bright light” most often triggered their migraine attacks. The mean (±SEM) length of time since the onset of migraine attacks was 16.0 ± 1.9 years, the mean estimated frequency of migraine attacks was 2.4 ± 0.4 per month, and the mean pain intensity of migraines as measured by the 6-point visual analogue scale was 3.8 ± 0.2. Although 23 of 39 were taking some form of daily medication (mostly the oral contraceptive pill; 15 migraineurs), none of the migraine subjects were taking prophylactic medication for migraine.

### Trigeminal nerve volume and maximum cross-sectional area analysis

**Fig. 2:**
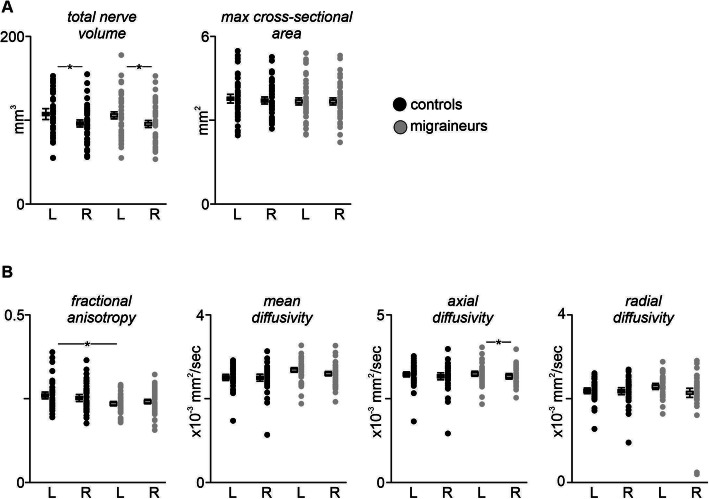
**a** Plots showing left and right nerve volumes and maximal cross-sectional areas in individual controls (black shading) and migraineurs (grey shading). Horizontal box plots indicate mean (±SEM) for the left and right nerves of each group. Note that only the volume was significantly different, i.e., lower in the right nerve for both controls and migraineurs. *p < 0.05. **b** Plots showing fractional anisotropy, mean diffusivity, axial diffusivity and radial diffusivity of the left and right trigeminal root in individual controls and migraineurs. Horizontal box plots indicate mean (±SEM) for the left and right nerves of each group. Note that only fractional anisotropy (FA) and axial diffusivity (AX) was significantly different, i.e., FA was lower in the left nerve of migraineurs than controls and AX was lower in the right nerve than the left nerve in migraineurs. *p < 0.05

**Fig. 3:**
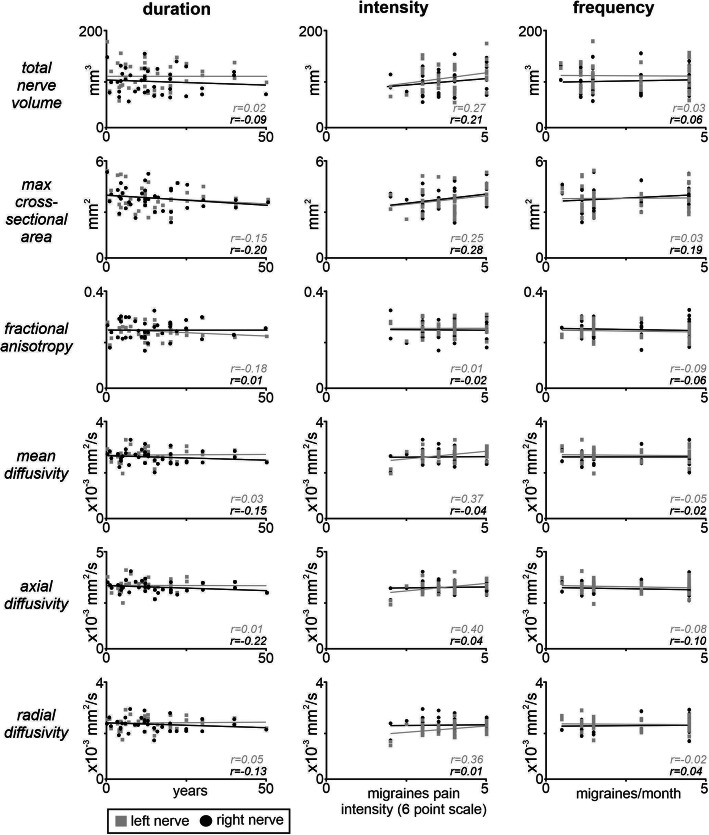
Plots showing linear relationships between the left (grey shading) and right (black shading) total nerve volume, maximum cross-sectional area, fractional anisotropy, mean diffusivity, axial diffusivity and radial diffusivity in migraineurs with migraine duration, intensity and frequency. Note that there were no significant linear relationships. *p < 0.05

Analysis of trigeminal nerve root entry zone volumes revealed that in both controls and migraineurs, total volume of the left nerve was significantly larger than that of the right nerve (controls: *p* < 0.001; migraine: p < 0.001) (Fig. 2a, Table 1). There was however no significant difference between left nerves in migraineurs compared with controls (*p* = 0.62) and similarly no difference between the right nerves (*p* = 0.79). In contrast, there were no significant differences between the left and right maximal cross-sectional areas in controls (*p* = 0.38) or migraineurs (*p* = 0.89) or between the left nerves (*p* = 0.41) or right nerves (*p* = 0.70) between groups. Additionally, there were no significant linear relationships between volume and either migraine duration (r = − 0.05, *p* = 0.77) or intensity (r = 0.26, *p* = 0.12) or frequency (r = 0.01, *p* = 0.94) or between maximal cross-sectional area and either migraine duration (r = − 0.19, *p* = 0.26) or intensity (r = 0.28, *p* = 0.08) or frequency (r = 0.12, *p* = 0.48) (Fig. 3). Furthermore, there were no significant linear relationships between the volume of the nerve ipsilateral or contralateral to the side of pain and either migraine duration (*ipsi:* r = − 0.17, *p* = 0.49; *contra:* r = 0.28, *p* = 0.25) or intensity (*ipsi:* r = 0.19, *p* = 0.44; *contra:* r = 0.44, *p* = 0.06) or frequency (*ipsi:* r = 0.11,*p* = 0.64; *contra:* r = − 0.08, *p* = 0.75) or maximal cross-sectional area and either migraine duration (*ipsi:* r = − 0.13, *p* = 0.58; *contra:* r = 0.12, *p* = 0.63) or intensity (*ipsi:* r = 0.38, *p* = 0.12; *contra:* r = 0.40, *p* = 0.09) or frequency (*ipsi:* r = 0.37, p = 0.12; *contra:* r = 0.28, *p* = 0.24). Intra-tracer reliability for global volumes had a mean Cronbach’s alpha of 0.98 (95%CI: 0.92, 0.99) and a mean ± SEM Dice similarity coefficient of 0.81 ± 0.04. Inter-tracer reliability for global volumes had a mean Cronbach’s alpha of 0.96 (95%CI: 0.85, 0.99) and a mean ± SEM Dice similarity coefficient of 0.80 ± 0.04.

**Table 1:** Overall mean (SEM) volume and maximum cross-sectional area of the trigeminal root entry zone in control and migraine subjects

	Controls		Migraineurs	
	*(n = 39)*		*(n = 39)*	
	*Left*	*Right*	*Left*	*Right*
**Total nerve volume (mm**^**3**^**)** mean (±SEM)	108.77 (3.97)*	96.32 (3.56)	105.86 (4.37)*	94.90 (4.05)
**Maximum cross-sectional area (mm**^**2**^**)** mean (±SEM)	3.81 (.12)	3.74 (.10)	3.67 (.12)	3.68 (.11)

Note that there was a significant difference between the left and right nerves of the control and left and right nerves of the migraine group. * *p* < 0.05, significant within group difference between left and right nerve volumes

### Trigeminal nerve diffusion analysis

One control and migraine subject were excluded from the trigeminal nerve diffusion analysis due to the absence of a clearly visible nerve boundary. Analysis of the remaining 38 subjects revealed significant differences between the left FA values of the controls and the left migraineurs (*p* = 0.01) with migraineurs displaying significantly reduced FA compared with controls (Fig. 2b, Table 2). In contrast, whilst in controls there were no significant differences in MD (*p* = 0.89), AX (*p* = 0.48), FA (*p* = 0.13) or RD (*p* = 0.93) between the left and right nerves, in migraineurs the magnitude of AX was significantly lower in the right nerve compared with the left (*p* = 0.04) with no significant differences in MD (*p* = 0.06), FA (*p* = 0.15) and RD (p = 0.06). However, overall, there were no significant differences between the left control and left migraineur values or between total MD (*p* = 0.15), AX (*p* = 0.85) or RD (p = 0.12) and right control and right migraineur values or between total MD (*p* = 0.67), AX (*p* = 0.73), FA (*p* = 0.21) or RD (*p* = 0.58) nerve volumes between controls and migraineurs. Additionally, there were no significant linear relationships between any of the four diffusion measures and either migraine duration (FA r = − 0.09, MD r = − 0.06, AX r = − 0.11, RD r = − 0.04) or intensity (FA r = − 0.01, MD r = 0.23, AX r = 0.26, RD r = 0.01) or frequency (FA r = − 0.08, MD r = − 0.03, AX r = − 0.11, RD r = 0.12) (Fig. 3). Finally, there were no significant linear relationships between any of the four diffusion measures of the nerve ipsilateral and contralateral to the side of pain and either migraine duration (FA *ipsi:* r = 0.17, *p* = 0.51; *contra:* r = − 0.28, *p* = 0.24; MD *ipsi:* r = − 0.36, *p* = 0.15; *contra:* r = − 0.01, *p* = 0.96; AX *ipsi:* r = − 0.38, *p* = 0.12; *contra:* r = − 0.06, *p* = 0.80; RD *ipsi:* r = − 0.19, *p* = 0.44; *contra:* r = 0.02, *p* = 0.93), migraine intensity (FA *ipsi:* r = 0.04, *p* = 0.88; *contra:* r = − 0.12, *p* = 0.66; MD *ipsi:* r = − 0.21, *p* = 0.41; *contra:* r = 0.29, *p* = 0.23; AX *ipsi:* r = − 0.22, *p* = 0.38; *contra:* r = 0.29, p = 0.24; RD *ipsi:* r = − 0.19, *p* = 0.45; *contra:* r = 0.29, p = 0.23), or migraine frequency (FA *ipsi:* r = − 0.06, *p* = 0.81; *contra:* r = 0.01, *p* = 0.97; MD *ipsi:* r = 0.23, *p* = 0.37; *contra:* r = 0.12, *p* = 0.64; AX *ipsi:* r = 0.21, *p* = 0.40; *contra:* r = 0.09, *p* = 0.70; RD *ipsi:* r = 0.30, *p* = 0.22; *contra:* r = 0.13, p = 0.60).

**Table 2:** Fractional anisotropy, mean diffusion, axial diffusion and radial diffusion values of the trigeminal nerve root entry zone in control and migraine subjects. Total nerve as well as caudal, middle and rostral third values are shown. * *p* < 0.05, significant within group difference between left and right nerve volumes. # *p* < 0.05, significant between group difference between left and right nerve volumes

	Controls		Migraineurs	
	*(n = 38)*		*(n = 38)*	
	*Left*	*Right*	*Left*	*Right*
**Fractional anisotropy** (mean [±SEM])				
Total nerve	.26 (.01)#	.25 (.01)	.23 (.01)	.24 (.01)
Caudal third	.24 (.01)	.24 (.01)	.22 (.01)	.23 (.01)
Middle third	.28 (.01)#	.27 (.01)	.25 (.01)	.26 (.01)
Rostral third	.25 (.01)*#	.24 (.01)	.22 (.01)	.22 (.01)
**Mean diffusion** (mean [±SEM] × 10^− 3^ mm^**2**^/sec)				
Total nerve	2.52 (.04)	2.51 (.05)	2.61 (.04)	2.54 (.04)
Caudal third	2.64 (.06)	2.62 (.06)	2.66 (.06)	2.67 (.06)
Middle third	2.44 (.04)	2.43 (.05)	2.53 (.05)	2.50 (.05)
Rostral third	2.59 (.05)	2.50 (.07)	2.70 (.05)	2.62 (.05)
**Axial diffusion** (mean [±SEM] × 10^−3^ mm^**2**^/sec)				
Total nerve	3.22 (.05)	3.17 (.06)	3.23 (.05)*	3.14 (.04)
Caudal third	3.26 (.07)	3.27 (.07)	3.25 (.07)	3.22 (.06)
Middle third	3.14 (.05)	3.12 (.06)	3.18 (.06)	3.11 (.05)
Rostral third	3.24 (.06)	3.13 (.08)	3.29 (.06)*	3.14 (.05)
**Radial diffusion** (mean [±SEM] × 10^−3^ mm^**2**^/sec)				
Total nerve	2.21 (.04)	2.19 (.05)	2.30 (.04)	2.23 (.04)
Caudal third	2.34 (.06)	2.34 (.06)	2.38 (.06)	2.35 (.06)
Middle third	2.09 (.04)#	2.09 (.05)	2.22 (.05)	2.14 (.05)
Rostral third	2.27 (.05)#	2.20 (.06)	2.42 (.05)*	2.27 (.05)

### Trigeminal nerve caudal, middle and rostral divisions analysis

**Fig. 4:**
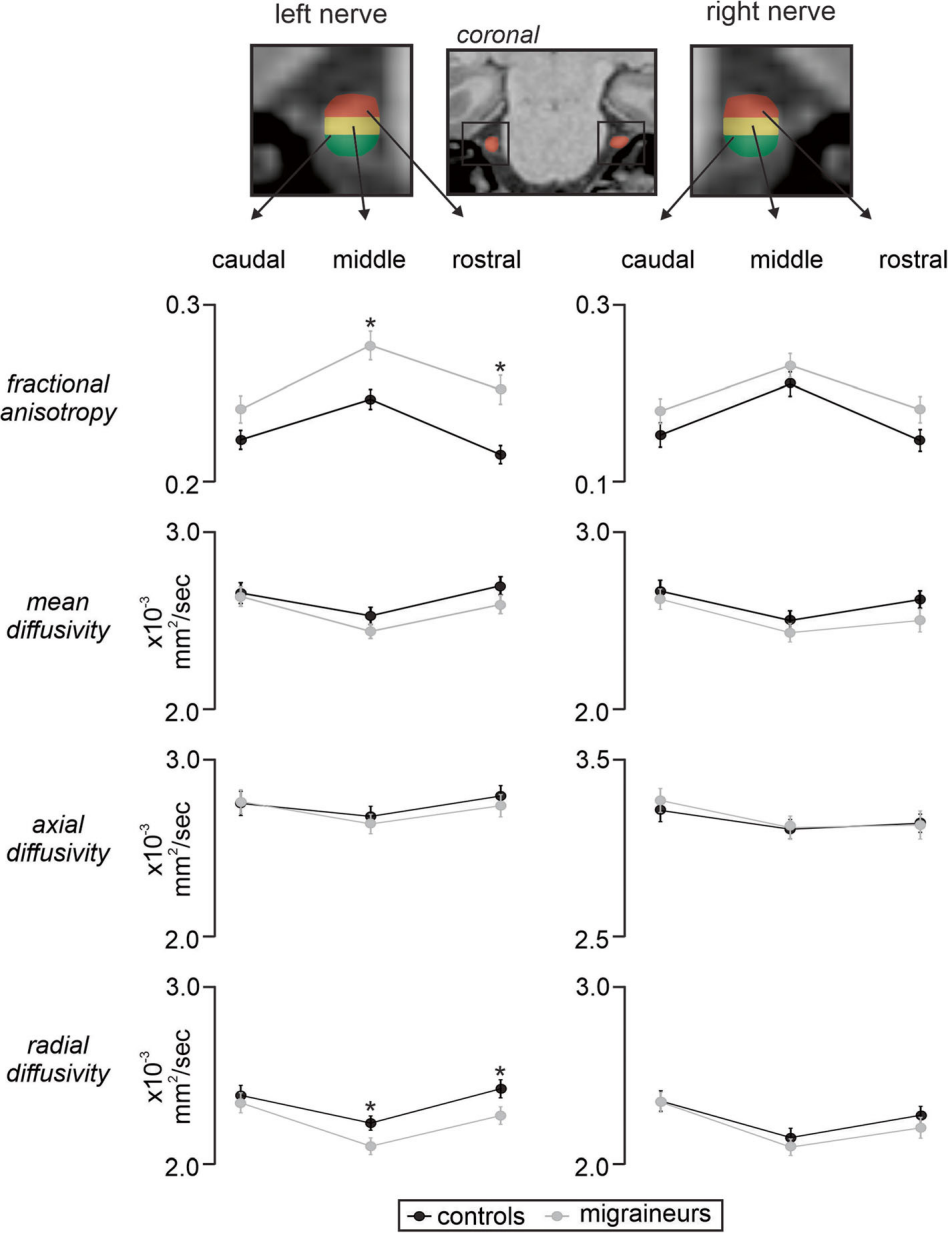
Line graphs showing mean (±SEM) fractional anisotropy, mean diffusivity, axial diffusivity and radial diffusivity of the left and right trigeminal root for caudal, middle and rostral divisions in controls (black lines) and migraineurs (grey lines). Note that fractional anisotropy was significantly different, i.e., lower in migraineurs than controls at the middle and rostral divisions, whereas radial diffusivity was greater in migraineurs at the middle and rostral divisions. *p < 0.05. The top panel shows a T1-weighted coronal image with the trigeminal nerve indicated by the red shading. To the left and far right is an expanded view of the left and right trigeminal nerve showing the caudal (green), middle (yellow) and rostral (red) divisions

Analysis of caudal, middle and rostral segments of the left and right trigeminal nerves revealed significant differences in FA with reduced FA in migraineurs compared with controls for the left nerve at the middle and rostral divisions (left FA: *caudal p* = 0.07; *middle p* = 0.003; *rostral p* = 0.0004; right FA: *caudal p* = 0.175; *middle p* = 0.279; *rostral p* = 0.077) (Fig. 4, Table 2). Furthermore, the left and right nerve analysis revealed a significantly greater RD value in migraineurs compared with controls for the left nerve at the middle and rostral divisions (left RD: *caudal p* = 0.61; *middle p* = 0.04; *rostral* p = 0.04; right RD: *caudal p* = 0.94; *middle p* = 0.44; *rostral p* = 0.36). In contrast, at no division was MD (left MD: *caudal p* = 0.81; *middle* p = 0.17; *rostral p* = 0.15; right MD: *caudal p* = 0.59; *middle p* = 0.37; *rostral p* = 0.16) or AX (left AX: *caudal p* = 0.92; *middle p* = 0.60; *rostral p* = 0.53; right AX: *caudal p* = 0.55; *middle* p = 0.92; *rostral* p = 0.92) significantly different between controls and migraineurs.

## Discussion

The results reveal that migraine is associated with reduced volume in the right nerve compared to the left nerve and reduced fractional anisotropy (FA) of the left trigeminal nerve root entry zone compared to controls. Whilst there were no overall differences in other diffusivity measures, radial diffusion (RD) was significantly greater in migraineurs at the middle and rostral parts of the nerve. In addition, we found no significant difference in maximal cross-sectional area between the left and right nerves of migraineurs and controls or between the groups. These data show that episodic migraine is associated with subtle changes in diffusivity that are consistent with changes in nerve microstructure, as revealed by previous histological analyses [1].

In contrast to our hypothesis, we found that the overall volume and cross-sectional area of the trigeminal nerve was not different in migraineurs compared with controls. This is unexpected as previous neuroimaging studies, including our own, have revealed increased or decreased trigeminal nerve volumes and cross-sectional areas in various orofacial pain conditions [2–5, 25]. Interestingly, the chronic orofacial pain conditions with reported changes in nerve volume are neuropathic in nature, whilst we have previously found that in individuals with the non-neuropathic chronic orofacial pain condition, painful temporomandibular disorder, the trigeminal nerve root entry zone displayed no difference in volume, cross-sectional area or diffusivity [5]. This likely reflects that temporomandibular disorder is maintained by constant nociceptor activation whereas neuropathic pain involves damage to the peripheral nerve itself [26]. It may also be true that given episodic migraine is an intermittent condition, in contrast with chronic orofacial pain that is usually unremitting pain, it has not elicited change in overall volume or cross-sectional area.

Interestingly, we also found a significant difference in total nerve volumes between the left and right sides in both controls and migraineurs. Controls and migraineurs had 11% and 10% smaller nerves on the right side compared with the left, respectively. Whilst not significant, we have previously shown the right trigeminal nerve root is on average 8% smaller than the left [5] and it has recently been reported that in healthy controls, the length of the trigeminal root entry zone is longer on the left compared with the right side [27]. In this study we found no difference in maximal cross-sectional areas between controls and migraineurs but differences in total volumes, which is consistent with differences in overall nerve length. It appears that whilst there are differences with respect to the levels of difference, overall, the right trigeminal nerve tends to be smaller than that of the left, even in healthy controls. Why this is the case is unknown, although it must be carefully considered when exploring trigeminal nerve differences, particularly in largely unilateral conditions such as trigeminal neuralgia.

While nerve volume is measured to provide a quantitative parameter of nerve damage, specific morphological changes can be difficult to investigate in living humans. However, underlying microstructural abnormalities indicative of a change in fiber content or orientation can be identified using diffusion weighted imaging. Whilst we found no overall change in mean diffusivity (MD) or axial diffusion (AX) in the nerve of migraineurs, we found a significant reduction in FA. As mentioned previously, FA is a scalar value between 0 and 1 that indicates the degree of anisotropy: a value of 0 means the diffusion in that voxel is unrestricted in all directions, whereas a value of 1 means that diffusion can only occur along one axis. FA is thought to be sensitive to changes in nerve myelination, axonal diameter or the organization of fibers, and the reduction in FA reported here in migraineurs is consistent with altered myelination and/or fiber organization [28].

A reduction in trigeminal nerve FA is consistent with a previous study which investigated histology of the zygomaticotemporal branch of the trigeminal nerve in 15 migraine patients and 15 matched controls [1]. Structural analysis revealed that migraine was associated with pathologic disruption throughout the zygomaticotemporal branch of the trigeminal nerve whereby the organization of a significant number of myelin sheaths and their target axons were disrupted. A differential distribution ranging from folded myelin constricting the axon to intact axons with thin myelin sheaths was observed, suggesting that not all axons are equally affected. Furthermore, the authors revealed that the characteristic wavy appearance of the longitudinal neurofilament expression, essential for establishment of normal axonal caliber, was discontinuous and poorly registered with the myelin sheaths. It was postulated that nerve abnormalities are suggestive of axonal abnormality and is consistent with our finding of a reduction in diffusivity along a single axis. Whilst we did not find an overall change in MD or RD in the trigeminal nerve of migraineurs, rostral-caudal analysis revealed that there was an increase in RD at the middle and rostral segments of the trigeminal nerve root entry zone. Although it is difficult to ascertain why in the more rostral segments RD increases whereas MD remains the same as controls, there is some evidence that FA and RD changes correlate with electrophysiological markers of demyelination, whereas MD does not [29]. Others have suggested that RD relates to myelin compactness [30]. Irrespective of the underlying pathology, our results are consistent with previous histological evidence and show that the microstructure of the trigeminal nerve is altered in migraineurs. Given the singular histological study exploring the zygomaticotemporal branch of the trigeminal nerve in migraine patients, further investigation is warranted to support the changes in DTI nerve diffusion parameters.

Although the overall change in diffusivity in migraineurs is consistent with our hypothesis, the rostral-caudal distribution of the changes was not. A previous study investigating the demography of sensory fibers in various orofacial pain conditions identified a greater ratio of myelinated *A*β- and *A*δ- fibers with less unmyelinated C- fibers in the rostral and middle portion of the nerve compared to the caudal region [8]. These findings suggest that microstructural changes in the rostral and middle segments of the trigeminal nerve in migraineurs likely reflect alterations in myelinated *A*β- and *A*δ- fibers. It has been argued that the headache phase of migraine results from activation of nociceptors in brain meninges and large cerebral arteries innervated by trigeminal afferents [31], which terminate in caudalis and interpolaris divisions of the spinal trigeminal nucleus as well as in the right upper cervical dorsal horn [32, 33]. Trigeminal afferents terminating in the spinal trigeminal nucleus are primarily C-fibers and we have recently shown reduced gray matter volume and increased diffusivity in this region in episodic migraineurs [20]. The increases in RD and the reduction in FA was restricted to the middle and rostral parts of the trigeminal nerve, suggesting that the microstructural changes do not occur in all fiber types of somatosensory afferents in migraineurs. Indeed, the diffusivity indexes of myelin anatomy were altered in the part of the nerve with myelinated axons and not in the caudal part which contains primarily unmyelinated axons. It has been postulated, however, that the cross-talk of CGRP signaling between adjacent C- and *A*δ- trigeminal nerve fibers demonstrated in a recent immunohistochemical study [12], may facilitate peripheral and central functions of nociceptive transmission in migraine which may further lead to trigeminal nerve aberrations evident in diffusivity changes.

Even though our data shows altered trigeminal nerve structure which would lead one to suggest that a peripheral trigger is necessary for migraine generation, this may not be the case. For many years, cerebrovascular changes have been considered the foundation of migraine initiation. While this might be true, others have suggested that migraine attacks are initiated by changes within one or more regions within the central nervous system, i.e., a central “migraine generator” [34, 35]. More specifically, migraine could result from dysfunction of subcortical sites leading to an “abnormal perception of basal level of primary traffic” [36]. Whilst the central generator theory of migraine remains controversial and has been the subject of fierce debate, our data are not incongruent with this idea. For example, it is possible that the microstructural changes that occur in the trigeminal nerve do not result from the effects of external triggers, but instead underlie altered basal input traffic which may allow a central event to more easily trigger a migraine attack. Furthermore, the treatment of migraine by botulinum toxin injections into a trigger site or transection of the zygomaticotemporal branch of the trigeminal nerve [37, 38] may be effective by reducing the basal afferent drive onto the spinal trigeminal nucleus. How the changes in trigeminal nerve structure in migraineurs relates to the initiation of migraine attacks or the commonly reported intracranial and extracranial mechanical hypersensitivities [39] remains unknown and warrants further exploration.

There are several limitations that need to be considered. This is a cross-sectional study and thus we cannot determine if the changes in diffusivity are dynamic in nature. A longitudinal study of patients to evaluate the prognostic value of the multiple diffusion tensor imaging (DTI) parameters and pain outcomes in migraine can determine whether nerve alterations in migraineurs are reversible and if structural integrity can be restored. In addition, we did not measure intracranial and extracranial mechanical hypersensitivities, which may be related to nerve structure. Interestingly, we found no significant linear relationships between any nerve diffusion parameter and migraine duration, intensity or frequency, suggesting that these three characteristics are not influenced by nerve anatomy. Finally, the spatial resolution of human DTI is relatively low and thus it is difficult to precisely localize each voxel of the trigeminal nerve. However, each nerve was initially defined from high-resolution T1-weighted anatomical images, where the boundaries are clearly visible. The relatively larger DTI voxel sizes would also increase the issue of partial-volume effects, although given that there was no significant overall volume difference between controls and migraineurs we suggest that this would not have been a significant factor in our analysis. Furthermore, future studies investigating nerve changes between the subgroup of migraineurs with aura and migraine without aura is warranted.

## Conclusions

Overall, our results suggest that migraineurs display microstructural changes in the trigeminal nerve, evident in-vivo. These changes may be reflective of demyelination or myelin compactness and corroborate previous ex-vivo histological findings. A greater understanding of the aberrant microstructure in the migraine trigeminal nerve may result in the development of non-invasive treatments for effective alleviation of migraine pain by targeting the anatomy of the trigeminal nerve or its branches.

## Data Availability

The datasets used and analyzed during the current study are available from the corresponding author on reasonable request.

## Abbreviations

MRI: Magnetic resonance imaging
CGRP: Calcitonin gene-related peptide
DTI: Diffusion tensor imaging
VOI: Volume of interest
FA: Fractional anisotropy
MD: Mean diffusion
AX: Axial diffusion
RD: Radial diffusion
